# An Exceptional Response to Dostarlimab in Mismatch Repair Deficient, Microsatellite Instability-High and Platinum Refractory Endometrial Cancer

**DOI:** 10.3390/curroncol29080413

**Published:** 2022-07-22

**Authors:** Michele Bartoletti, Giorgio Giorda, Alessandra Viel, Mara Fornasarig, Adrian Zdjelar, Enrica Segatto, Roberto Sorio, Serena Corsetti, Simona Scalone, Milena Sabrina Nicoloso, Tania Pivetta, Emilio Lucia, Nicolò Clemente, Elisa Palazzari, Vincenzo Canzonieri, Fabio Puglisi

**Affiliations:** 1 Unit of Medical Oncology and Cancer Prevention, Department of Medical Oncology, Centro di Riferimento Oncologico di Aviano (CRO), 33081 Aviano, Italy; rsorio@cro.it (R.S.); serena.corsetti@cro.it (S.C.); sscalone@cro.it (S.S.); mnicoloso@cro.it (M.S.N.); tania.pivetta@cro.it (T.P.); fabio.puglisi@cro.it (F.P.); 2 Unit of Gynecologic Oncology Surgery, IRCCS CRO Aviano, National Cancer Institute, 33081 Aviano, Italy; ggiorda@cro.it (G.G.); elucia@cro.it (E.L.); nicolo.clemente@cro.it (N.C.); 3 Unit of Functional Oncogenomics and Genetics, Centro di Riferimento Oncologico di Aviano (CRO) IRCCS, 33081 Aviano, Italy; aviel@cro.it; 4 Unit of Oncologic Gastroenterology, Centro di Riferimento Oncologico di Aviano (CRO) IRCCS, 33081 Aviano, Italy; mfornasarig@cro.it; 5 Department of Oncologic Radiation Therapy and Diagnostic Imaging, Centro di Riferimento Oncologico, Via Franco Gallini, 2, 33081 Aviano, Italy; adrian.zdjelar@cro.it (A.Z.); esegatto@cro.it (E.S.); 6 Department of Medicine, University of Udine, 33100 Udine, Italy; 7 Radiation Oncology, Centro di Riferimento Oncologico di Aviano (CRO) IRCCS, 33081 Aviano, Italy; elisa.palazzari@cro.it; 8 Pathology Unit, Centro di Riferimento Oncologico di Aviano (CRO) IRCCS, 33081 Aviano, Italy; vcanzonieri@cro.it; 9 Department of Medical, Surgical and Health Sciences, University of Trieste, 34127 Trieste, Italy

**Keywords:** endometrial cancer, dostarlimab, immunotherapy, predictive biomarkers

## Abstract

Until recently, effective therapies for advanced endometrial cancer progressing to a platinum-based combination were lacking. In this setting, immunotherapy with anti PD-1/PDL-1 monoclonal antibodies is rising as a new paradigm in particular for patients with microsatellites instability/mismatch repair deficiency. In this case report, we describe an exceptional and rapid response to dostarlimab in a platinum refractory endometrial cancer patient with high disease burden harboring a mismatch repair deficiency.

## 1. Introduction

Endometrial cancer (EC) represents a public health concern as its incidence and mortality are raising worldwide [1]. Even if in most cases it is diagnosed at early stage where cure rates are high, about 10% of EC cases are de novo metastatic or relapse after surgery and adjuvant therapies [2]. For patients with advanced EC, effective therapies beyond platinum salts are lacking, and no standard chemotherapies nor targeted agents have been recognized until recently. In fact, the high percentage of about 30% of patients with mismatch repair deficient (dMMR)/microsatellite instable (MSI) or POLE mutant EC has paved the way to immunotherapy, and in August 2021, the Food and Drug Administration (FDA) has granted accelerated approval for the anti-PD1 dostarlimab in recurrent dMMR/MSI EC, based on a phase I trial [3,4].

Despite more solid data from phase III clinical trials being awaited, dostarlimab is currently used in dMMR/MSI EC patients who have no satisfactory alternative treatment options. Here, we report a case of a platinum refractory dMMR/MSI EC who gained a rapid, long-lasting, and near-complete radiological response with second-line dostarlimab.

## 2. Case Report

A 78-year-old female was referred to the National Cancer Institute in Aviano for EC diagnosed in 2018 when she underwent radical hysterectomy with pelvic lymphadenectomy for FIGO stage I, high-grade endometrioid adenocarcinoma with substantial lymphovascular space invasion. The risk factors suggested the need of adjuvant chemoradiation with systemic carboplatin-paclitaxel and external beam radiotherapy completed in May 2019. For back pain in the lumbar region, a computed tomography (CT) scan was performed in September 2020, showing an interaortocaval lymphadenopathy of 22 mm.

A second-level PET reinforced the suspicion of recurrent EC, and in December 2020, a secondary surgery with paraaortic lymphadenectomy was performed without evidence of residual disease at post-surgical imaging. The patient was in good condition, and she received first-line chemotherapy with six cycles of carboplatin AUC 5 and paclitaxel 175 mg/m^2^. Prior to starting follow-up, a CT scan was performed in June 2021, unveiling disseminated nodules in both lungs and three large liver metastases of 68 mm, 57 mm, and 40 mm (Video S1).

The patient was still in good performance status but with mild and sporadic cough in bona fide related to the tracheobronchial disease’s involvement. The tumor specimens from the first and second surgeries were retrospectively analyzed for the expression of the mismatch repair proteins and showed no staining for MLH1 and PMS2. Tumor DNA sample was analyzed (OncoMate MSI DX Analysis system, Promega) and high genetic instability in five out of five microsatellites was found. The patient was referred to a geneticist, and a DNA methylation test (MS-MLPA) performed on tumor tissue discovered somatic MLH1 gene promoter hypermethylation.

The patient was informed about the lack of effective standard therapies and accepted reception dostarlimab in the expanded access program active in Italy. She started dostarlimab in August 2021 at 500 mg every 3 weeks and then 1000 mg every 6 weeks from cycle 4. Treatment was well tolerated without any adverse effects, while the mild cough vanished in about 3 to 4 weeks.

The first radiological evaluation (11 weeks from treatment’s started, images not presented) performed in October 2021 showed a nearly complete radiological response on both lungs and partial response in the liver where the metastases were reduced in size and necrotizing. At the following CT scan (6 months from treatment’s started), lung lesions had almost disappeared, and the liver metastases were reduced to 34 mm, 25 mm, and 14 mm, respectively (Video S1).

At the most recent assessment, in May 2022 (9 months after the start of treatment), the patient is still on therapy with dostarlimab 1000 mg every 6 weeks in complete clinical response and without adverse events or residual toxicities.

## 3. Discussion

Platinum salts are the most active drugs for gynecological malignancies. For platinum-resistant or -refractory disease (i.e., disease progression within 6 months or during platinum-based therapy), available options are dismal in terms of activity, and patient’s prognosis is poor. Here we report a case of platinum-refractory dMMR/MSI EC effectively treated with dostarlimab with a complete radiological response.

Recently, standard chemotherapies and endocrine therapy were the only treatment options for recurrent endometrial cancer and predictive biomarkers with match targeted drugs were lacking.

Even for early-stage disease, adjuvant therapies were tailored based on classic pathological features such as histotype, tumor grade, myometrial and lymph node involvement, and lympho-vascular space invasion. Molecular analysis at a genomic level has redefined the classification of endometrial cancer, unveiling four prognostic molecular subtypes known as ultramutated (POLE mutant), hypermutated (microsatellite instable), copy-number low (not otherwise specified), and copy-number high or serous-like (TP53 aberrant) [5]. In recurrent or advanced disease, tumor molecular profiling is increasingly important since several biomarkers are influencing the treatments’ algorithm and several others are coming.

In fact, HER2 expression is currently tested in patients with serous EC, as nearly 25% of cases overexpress the proteins and seem to benefit from the addition of trastuzumab to platinum-based chemotherapy [6]. Even hormone receptors (estrogen and progesterone) are used as predictive markers for endocrine therapy with medroxyprogesterone acetate or megestrol acetate in low-grade tumors [7]. Recently, dostarlimab has shown a remarkable response rate in advanced EC, especially in those harboring dMMR/MSI alteration.

Although this evidence derives from a multi-cohort phase I study, the exceptional activity of this anti-PD1 antibody has gained conditional marketing authorization from EMA in April 2021 for dMMR/MSI endometrial cancer who progressed on or after a platinum-based chemotherapy [8]. As a matter of fact, in the GARNET trial in 71 patients with measurable disease, there was a confirmed response in 30 cases (objective response rate of 42.3%; 95% CI, 30.6–54.6%). In nine cases, a complete response was registered (12.7%). Responses were long lasting with more than 75% progression-free patients at 12-months. Anemia (2.9%), colitis (1.9%), and diarrhea (1.9%) were the most common grade 3 adverse events [4]. In this setting, the anti-PD1 antibody pembrolizumab has also showed similar results in the multi-cohort KEYNOTE-158 trial [9]. Based on data showing an ORR of 46% (95% CI, 35–56%), the FDA has approved single-agent pembrolizumab in March 2022 [10].

These and others promising data have led to two potential practice-changing trials for dMMR/MSI EC where dostarlimab and pembrolizumab are compared to platinum-based chemotherapy in a first-line setting (NCT05173987, NCT05201547). Moreover, the second line scenario is enriched by the recently published results of the KEYNOTE-775 trial. This phase III study has established the combination of pembrolizumab and lenvatinib the new standard of care in patients previously treated with platinum salts [11]. In this trial, the immune-based combination was compared to standard chemotherapy at investigator’s choice in 827 patients (of which only 130 were dMMR). Both the primary outcomes of progression-free and overall survival were in favor of the experimental arm, with more than 5 months of gain in OS in all comers. Grade ≥ 3 toxicities were reported in nearly 90% of patients receiving lenvatinib-pembrolizumab (mainly hypertension, weight decrease, and diarrhea) [11].

Whether dMMR/MSI patients could benefit enough from single-agent anti-PD1, sparing the toxicity of combination therapy, is still un unsolved question. Patient selection could be the key in the treatment choice between single-agent anti-PD1 or immune-combination, and in the near future, factors such as tumor mutational burden and patient’s microbiome characteristics might better identify dMMR/MSI-H patients who could benefit from a treatment intensification. Even the potential predictive role of pathogenic mutations in genes such as *PIK3CA*, *PTEN*, *ARID1A*, and *FGFR* that are frequently mutated in EC should be explored.

## Data Availability

The data presented in this study are available on request from the corresponding author.

## Supplementary Materials

The following are available online at https://www.mdpi.com/article/10.3390/curroncol29080413/s1, Video S1: Computed tomography scan shows tumor burden at baseline (June 2021) and at second radiological evaluation (6 months after treatment’s starts) with a complete response in lungs.
